# Enlarge the Training Set Based on Inter-Class Relationship for Face Recognition from One Image per Person

**DOI:** 10.1371/journal.pone.0068539

**Published:** 2013-07-16

**Authors:** Qin Li, Hua Jing Wang, Jane You, Zhao Ming Li, Jin Xue Li

**Affiliations:** 1 College of Physics Science and Technology, Shenzhen University, Shenzhen, China; 2 Department of Computing, The Hong Kong Polytechnic University, KLN, Hong Kong; 3 Lab of Medical Devices, Shenzhen Academy of Metrology and Quality Inspection, Shenzhen, China; Universidad de Castilla-La Mancha, Spain

## Abstract

In some large-scale face recognition task, such as driver license identification and law enforcement, the training set only contains one image per person. This situation is referred to as one sample problem. Because many face recognition techniques implicitly assume that several (at least two) images per person are available for training, they cannot deal with the one sample problem. This paper investigates principal component analysis (PCA), Fisher linear discriminant analysis (LDA), and locality preserving projections (LPP) and shows why they cannot perform well in one sample problem. After that, this paper presents four reasons that make one sample problem itself difficult: the small sample size problem; the lack of representative samples; the underestimated intra-class variation; and the overestimated inter-class variation. Based on the analysis, this paper proposes to enlarge the training set based on the inter-class relationship. This paper also extends LDA and LPP to extract features from the enlarged training set. The experimental results show the effectiveness of the proposed method.

## Introduction

Face recognition has attracted much attention in the last two decades. However, it is still an unsolved problem that needs further investigation. Several factors challenge the current face recognition techniques, including the variations of pose, illumination, expression, age, and the occlusion. Face recognition from one image per person (also referred to as one sample problem) is another important sub-area, which recently attracts increasing attention [1]. One sample problem is particularly significant in some large scale identification problems, such as passport card identification, driver license identification, and law enforcement.

The most popular face recognition methods are subspace-based methods, including principal component analysis (PCA) [2], Fisher linear discriminant analysis (LDA) [3], locality preserving projections (LPP) [4], and so on. The subspace-based methods first seek a set of projection vectors and then project the original image onto these projection vectors. With several training images per person, the subspace-based methods achieved high classification accuracy. However, their performances degrade significantly as the number of training images decreases. The task of face recognition from one image per person is an extreme situation where we have the fewest training images. Many popular subspace-based feature extraction methods [2]–[6] and classifiers [7]–[11] either cannot achieve high classification accuracy, or fail to work in one sample problem.

Researchers have proposed methods to deal with one sample problem. The extensions of PCA [12]–[13] fade out the unimportant features in a preprocessing procedure before performing PCA. By incorporating prior information of the within-class scatter from other people, Wang et al. [14] solve one sample problem based on the assumption that human being exhibits similar intra-class variation. There are also some methods [15]–[19] that can enlarge the training set and turn the one sample problem into multiple samples problem. While the methods [12]–[19] mainly focus on making the conventional methods applicable to one sample problem, they do not present the reasons that make one sample problem difficult.

In this paper, we analyze why face recognition is difficult from two different viewpoints. The first viewpoint is the principal of the popular feature extraction methods. We study the principals of PCA, LDA, and LPP and show why they cannot perform well or applicable to one sample problem. We also present our analysis from the second viewpoint: why is one sample problem itself difficult? For the first time, we ascribe the difficulty of one sample problem to four reasons: 1. the training set is small; 2. one sample is not representative; 3. the intra-class variation is unknown or underestimated; and 4. the inter-class variation is overestimated.

Our analysis leads us to solve the one sample problem by enlarging the training set based on the inter-class relationship. By synthesizing many samples, our method not only turns the one sample problem into a multiple samples problem, but also can rectify the underestimated intra-class variation and the overestimated inter-class variation. In the enlarged training set, the synthesized images for one individual are independent from each other. This enhances the representative of the training set. We propose extensions of both LDA and LPP for feature extraction from the enlarged training set. These two extensions treat the real images and the synthesized images differently, and suitable for use on the enlarged training set. The experimental results show that the feature extraction methods achieve higher classification accuracy on the enlarged training set.

## Background

PCA, LDA, and LPP are three popular methods proposed for feature extraction in the task of face recognition. These three methods and their extensions are developed based on an implicit assumption that several images (at least two) from each individual are available in the training stage. As this implicit assumption does not hold in the one sample problem, these methods cannot achieve high classification accuracy. In the following, we analyze why one sample problem degrades the performances of PCA, LDA, and LPP in face recognition.

As one of the most popular methods, PCA (also known as Eigenfaces [2]) seeks a set of projection vectors that can maximize the total scatter matrix. The low-dimensional representations in PCA are most representative and have minimum reconstruction error. Mathematically, PCA maximizes the total scatter matrix 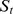.
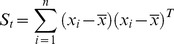
(1)


It is proved that the total scatter matrix can be rewritten as [5]

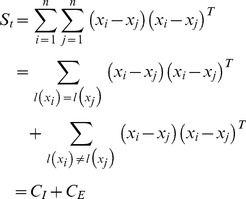
(2)where 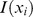 is the label of sample 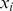. Equation (2) shows that the total scatter matrix contains both the intrapersonal subspace and extrapersonal subspace [5]. With one training image per person, the first term 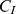 corresponding to the intrapersonal subspace equals zero and the total scatter matrix only contains the extrapersonal subspace. It seems that maximizing only extrapersonal subspace is better for recognition. However, this is true only in the cases where the capture conditions of the testing and training face images are the same or at least similar, and subject to few variations of illumination, pose, and expression. Though the total scatter matrix can capture the major identification difference among training face images, they fail to do so when the testing face images are captured under different conditions [5]. This is justified by the fact that the accuracy of PCA drops more than 30% when the number of training face images for each individual drops from 9 to 1 [1].

LDA (known as Fisherfaces [3]) aims to maximize the inter-class variation and simultaneously minimize the intra-class variation. In one sample problem, as no pair of face images shares the same class label, intra-class variation is unknown and the intra-class scatter matrix is zero. Because the projection vector does not change the null intra-class scatter matrix, the LDA-based projection vectors are the ones that maximize the inter-class scatter matrix in one sample problem. In other words, LDA degenerates to PCA in one sample problem.

LPP (known as Laplacianfaces [5]) seeks representations of the face images that preserve most local structure. In the LPP, two face images should be near to each other in the feature space if they are neighbors in the original image space. If the face images of each individual respectively cluster together, this method can generate low dimensional representations for them with high separability. In one sample problem, however, the local structure is rarely useful for classification as the neighbor face images associate with different individuals. Thus, LPP which heavily relies on the local structure cannot perform well in one sample problem.

### Why is One Sample Problem Difficult?

From the viewpoint of feature extraction principal, above section analyzed why three popular methods cannot perform well in one sample problem. These analyses summarize and extend the analyses in [1], [12], [20]–[23]. In the following, we will present our analysis from a new viewpoint: why is one sample problem itself difficult? Based on our understanding, the one sample problem is difficult mainly due to the following four reasons.

Firstly, the task of face recognition is essentially a small sample size (SSS) problem, and one sample problem is the extreme situation. The face images are normally of tens of thousands of dimensional. By contrast, the number of available face images for each individual is normally much smaller, and decreases to its minimum value in one sample problem. It is proved that if the samples are of 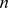 dimensional, we need 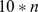 samples to learn a robust model [24]. The training samples are far from enough in the task of face recognition and the SSS problem occurs. Thus, face recognition is essentially a SSS problem. The dilemma between the high dimension and the small sample size is even more serious in one sample problem.

Secondly, one image is not representative enough in the task of face recognition. It is widely recognized that the variations of pose, illumination, expression can induce large variations on the face images. Face images of the same individual are different from each other if they are captured under different conditions. As the capture environment changes, the difference among face images from the same individual is not avoidable. One image is far from enough to represent the face images of one individual. Researchers have studied the relationship among face images captured under different conditions and found ways to predict on from the others [16], [25]–[26]. In the training stage of multiple samples problem, not only the available face images can be directly used but also the latent ones that are predictable from the training images can be indirectly used. For example, if we have two face images of one individual where one image with frontal pose and one image with pose variation of 15 degree to the left. We can easily obtain the image with pose variation of 15 degree to the right. From a single image, however, it is difficult to know how the face images will vary when condition changes and to predict images captured under novel conditions. In other words, we can rely on the synthesized images (based on intra-class relationship) in multiple samples problem, but cannot rely on them in one sample problem. To sum up, compared with multiple samples problem, one sample problem not only provides fewer samples but also offers less opportunity to use the latent samples.

Thirdly, as the intra-class variation is unknown, one samples problem deprives the opportunity of feature extraction methods to minimize the intra-class distance, and provides far from enough inputs for classifiers in the training stage. To achieve high classification accuracy, most feature extraction methods in pattern recognition try to minimize the intra-class distance in the feature space. However, the intra-class variation is unavailable in one sample problem. This deprives our chance to minimize the intra-class variation in the feature extraction procedure. Thus, the intra-class variation is large with high probability in the feature space and unfavorably affects the following classification procedure. We need the inter-class and intra-class variation to train classifiers [7]–[8]. The classifiers classify a testing sample based on its relationship to the training samples. If the difference between a testing and training sample is considered to be intra-class variation, the classifier labels the testing sample using that of the training sample. As the intra-class variation is not available in the one sample problem, we cannot train a robust classifier.

Fourthly, the inter-class variation is overestimated in one sample problem. The inter-class variations measure the differences between images that have different class labels. As there is only one image per person in one sample problem, all the variations are inter-class variations. The following analysis shows how the inter-class variation is overestimated.

**Figure 1:**
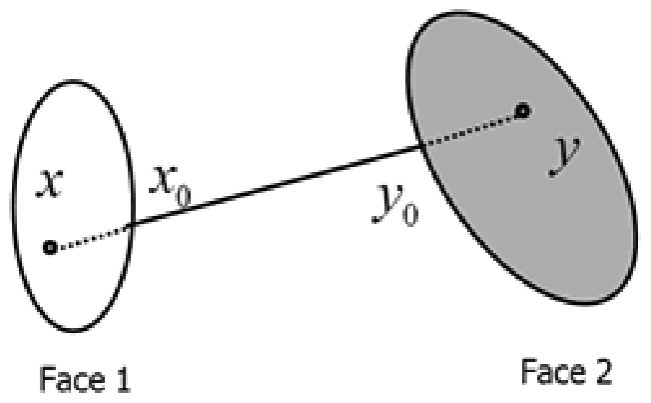
The overestimated inter-class variation.

We suppose the face images of two individuals respectively form a cluster, as shown in figure 1. In figure 1, the two ellipses represent two clusters respectively formed by the images of face 1 and face 2. The training image 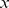 is from face 1 and 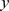 is from face 2. The difference between these two face images 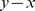 is considered as an inter-class variation in one sample problem. In fact, as can be seen from figure 1, 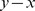 is much larger than the true inter-class variation. Assume 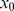 and 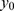 are two latent images locate on the intersections of ellipses and the line that joints 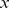 and 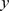. The estimated inter-class variation 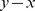 is consists of three sections: the intra-class variation of face 1, i.e. 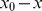; the intra-class variation of face 2, i.e. 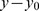; and the real inter-class variation, i.e. 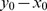. The inter-class variation is supposed to be maximized in feature extraction methods. When feature extraction methods maximize such an overestimated inter-class, they exaggerated the intra-class variations of face 1 and face 2 at the same time. This degrades the performance of the classification procedure.

From the above analysis, we conclude that the difference between the one sample problem and multiple samples problem is beyond the number of training samples. It is the above four reasons that make one sample problem more difficult.

## Proposed Methods

In this section, we will propose a novel method to enlarge the training set based on the inter-class relationship.

### 1 Basic Idea

We consider the face images as points in the high dimensional face space. Due to the variations of pose, illumination, and expression, face images of the same individual are different from each other and represented by different points. However, as they associate with the same individual, these images have some similarity to each other and the corresponding points form a cluster. This is especially true when the capture environment does not change significantly. Based on this observation, we assume that the images of one individual cluster together in this paper, as shown in figure 1.

Regarding the image 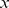 from face 1 and 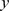 from face 2 as two points in the face space, we can use a line segment to joint them. This line segment consists of a series of points, each of which represents a latent image. This line segment can be represented by the following formula
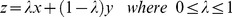
(3)


Note that, it is not necessarily that all of these points are real images. The points in the middle of this line segment are far from both of the real images and they are not real images in most cases. However, having small differences to one of the real images, the ones near to the end points can be considered as variations of the real images.

### 2 Image Synthesis

To synthesize images using (3) based on two images 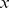 and 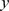, we need to fix the parameter 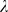. This paper confines this parameter into the union of two sets 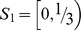 and 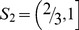. If 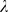 takes a value in 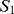, equation (3) synthesizes a variation for 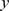 ; if 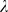 takes a value in 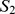, equation (3) synthesizes a variation for 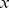. Here, we consider 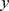
(or 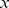) as an image synthesized using (3) when the parameter 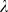 equals to 0 (or 1). In the set consists of the original images and the ones synthesized using (3), we can prove that the intra-class variation is smaller than the inter-class variation in terms of Euclidean distance, as follows:

Proof.

Suppose two images 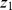 and 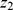 are synthesized using (3) respectively corresponding to parameter 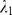 and 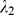, as follows
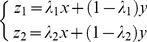
(4)


The distance between them can be computed
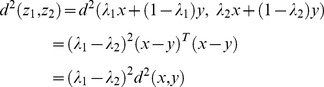
(5)


a). If 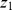 and 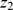 are synthesized images for the same image 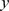 (or 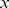), both 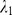 and 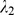 are from the same set 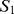 (or 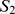). In this set 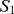 (or 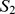), the difference between these two parameters is smaller than1/3. Thus,
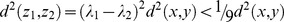
(6)


b). If 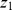 and 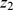 are synthesized images for two different images, 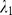 and 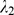 are from two different sets 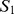 and 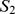. Thus, the difference between these two parameters is larger than 1/3, and
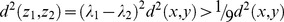
(7)


Based on a) and b), we know that all the intra-class variations are smaller than 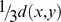 and all the inter-class variations are larger than 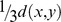. Thus, the intra-class variations are smaller than the inter-class variations. This ends the proof.

In the above, we talk about the image synthesis based on two images. In a multi-class problem, however, we must take more into consideration to obtain small intra-class variations and large inter-class variation. We design the following algorithm for face image synthesis in a multi-class problem:

Algorithm 1.

For each real image 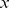, the following two steps synthesize its variations:

Step 1: among all the real images, find 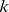 nearest neighbors of 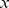 and denote them as 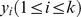, where 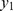 is the nearest neighbor;

Step 2: synthesize images using 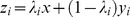, where 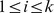 and 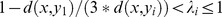


Using the above algorithm, we can synthesize many images to enlarge the training set. This training set has two properties.

Firstly, a image 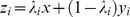 synthesized in step 2 is nearer to 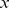 than to any real face image different from 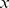.

Proof:

Suppose 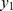 is the nearest neighbor of 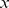 among all the real images, then we have the following formula
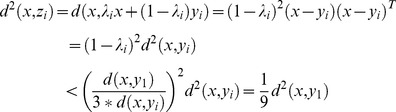
(8)


Thus,
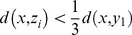
(9)


Suppose 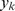 is a real image different from 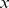, then
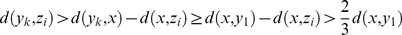
(10)


Based on (9) and (10), we know that the synthesized image 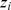 is much nearer to the real image it associating with than to the other real images.

Secondly, if 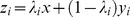 is a variation of 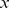 and 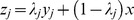 is a variation of 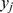, then 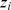 is nearer to 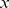 than to 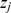, i.e. 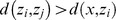.

Proof:

Based on the triangle inequality theorem, we know that
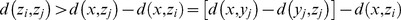
(11)


Based on (9), we have
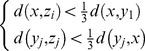
(12)


Thus,
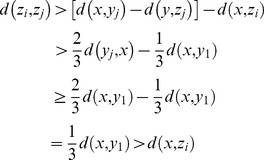
(13)


So,
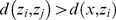
(14)


### 3 Discussion

We can use algorithm 1 to synthesize variations for each real face image and obtain an enlarged training set. This enlarged training set has four properties.

Firstly, this set of images has a reduced intra-class variation and increased inter-class variation. As mentioned above, the intra-class variation is underestimated and the inter-class variation is overestimated in one sample problem. We can easily prove that, compared to 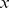, 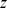 has a smaller distance to 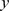, if 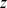 a variation of 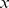 synthesized based on (3). For some 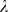, the synthesized variation can equal to 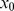 or 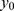 that locates on the margin of the area for a face (shown in Figure 1). Though the synthesized variation is usually not the exactly samples on the margin, they are usually near to them. Through this way, the estimated inter-class variation is more accurate. As we divide the original inter-class variation (the difference between 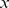 and 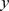) into three portions (one reduced inter-class variation and two intra-class variations), we increase the intra-class variation and reduce the inter-class variation. Also, what the intra-class variation is increased is what the inter-class variation is reduced. With the intra-class variation, we have an opportunity to minimize it in the feature extraction procedure.

Secondly, the local structure is useful for classification in the enlarged training set. It is proved that the synthesized samples are nearer to the real face images belonging to the same individual than the real face images of the others. In other words, each image must have a neighbor that share the same class label with it. Because of this, the feature extraction method that keeps the local structure will generate a small intra-class variation in the feature space. Thus, the local structure is useful for classification.

Thirdly, the enlarged training dataset makes it possible to learn a robust model for feature extraction. If we synthesize 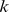 variations for each of the real face images, the enlarged training set will be 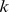 times larger than the original training set. With the training set consists of 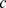 images from 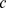 individuals, the largest enlarged training set consists of as many as 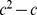 images. In other words, the largest enlarged training set is nearly quadratically larger than the original training set. This alleviates the dilemma between high dimensionality and small sample size.

Fourthly, the synthesized images captured the variations along different directions. Step 2 synthesizes images based on an image and its several neighbors, which are normally along different directions. This enriches the variations of the training set and enhances its representation. Also, the synthesized images are independent if they are synthesized based on different pairs of real images.

## Extensions of LDA and LPP for Dimension Reduction

In this section, the 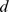 dimensional vector 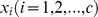 represents the image from the 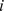
th individual. In all, we have 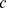 real images from 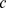 individuals. To enlarge the training set, we use algorithm 1 to synthesize variations for these real images. The 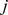
th synthesized image for the 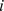
th individual is represented by 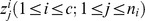, where 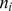 represents the number of images synthesized for the 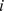
th individual. Thus, the training set consists 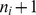 samples for the 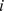
th class, including one real image and 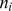 synthesized images. The total number of the synthesized images is 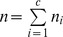. In the following, we propose extensions of LDA and LPP for dimension reduction.

### 1 LDA Extension

LDA aims to maximize the inter-class variation and simultaneously minimize the intra-class variation. The projection vectors are obtained by maximizing the following Fisher criterion
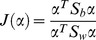
(15)where 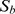 and 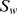 respectively represents the inter- and intra-class scatter matrix. These two matrices are popularly defined as follows
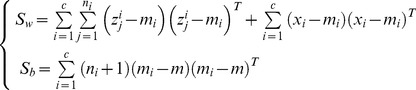
(16)


where 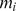 and 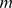 represent the mean of the 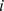
th class and the whole training set, respectively.

In this one sample problem, we take the real image as the mean of the 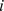
th class, and compute the intra-class scatter matrix as follows
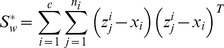
(17)


Though the synthesized images are neighbors of the real images, it is possible that they do not accurately model the variations of the real image. The mean computed based these synthesized images may vary from the real mean value. It is reasonable to take the real image as the mean value. Through this way, we not only save the time to compute the mean value, but also alleviate the adversely effect (if any) of the synthesized images. Even if the synthesized images do not accurately model the variations of the real image, we still can get the valid mean value of the 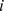
th class.

We can rewrite the inter-class scatter matrix as follows
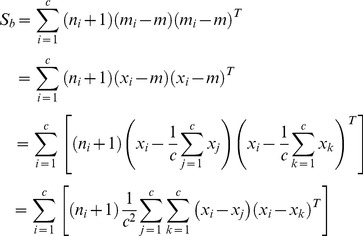
(18)


Equation (18) shows that the matrix 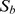 is derived based on the differences between the real images. As mentioned above, the difference between the real images overestimated the inter-class variations. Thus, the inter-class scatter matrix is not accurately estimated. We newly define the inter-class scatter matrix as follows
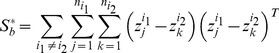
(19)


This inter-class scatter matrix is derived based on the differences between the synthesized images. Based on our discussion, such differences model the inter-class variations more accurately.

To summary, we seek LDA-based projection vectors by maximizing the following Fisher criterion
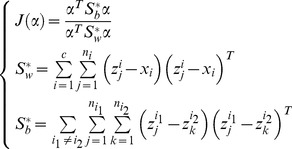
(20)


The feature extractors that maximize the above Fisher criterion are the eigenvectors of the following generalized eigen-equation problem corresponding to the maximum eigenvalues
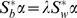
(21)


### 2 LPP Extension

LPP tries to learn a subspace that preserves the local structure of the image space. In this paper, we propose the following extension of LPP for one sample problem
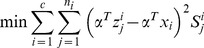
(22)


We define 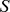 as follows
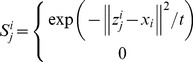
(23)where the positive 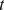 is sufficiently small, and it defines the radius of the local neighborhood. The objective function is different from the conventional one. If all the training samples are represent by 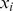, the conventional object function is defined as follows [4]

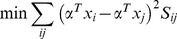
(24)where



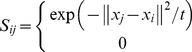
(25)In (22), we only consider the intra-class relationship between the real images and their synthesized variations. The relationship between the real images of different individuals and the synthesized images of different individuals are neglected. The reason behind doing this is the previously proved observation: the synthesized images 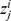 are near to the real image 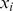. The physical meaning of (22) is as follows: the representations of the synthesized images 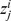 are expected to be neighbors of that of the real image 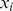 in the feature space.

To solve the optimization problem (22), we have the following steps
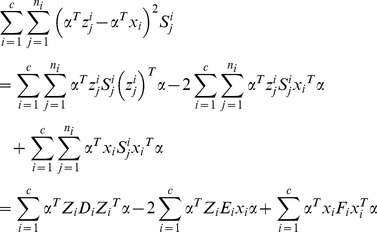
(26)


where 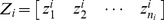
consists of all the synthesized images of the 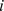
th class, 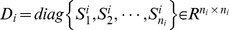, 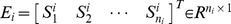, and 
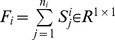
. Equation (26) can be further simplified to be
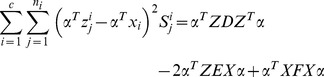
(27)where 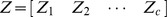 consists of all the synthesized images, 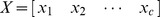 consists of all the real images, 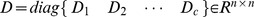, 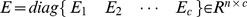, and 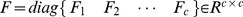.

We introduce a constraint as follows
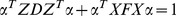
(28)


The minimization problem (24) reduces to
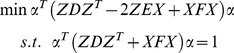
(29)


Based on (29), the projection vectors are the eigenvectors of the following generalized eigenvalue problem corresponding to the minimum eigenvalue
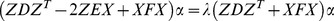
(30)


## Experiments

The ORL [27] is one of the most popular face image databases. This database contains ten face images each for forty different people. In order to provide suitable research material, the images of this database were taken at different times, and in various lighting. To model the faces in daily life, the faces had different expressions (open/closed eyes, smiling/not smiling) and some of them were facilitated with details (glasses/no glasses).

The Yale database [28] contains totally 165 images, 11 images from each of 15 individuals. The images have variations in lighting conditions facial expressions (normal, sad, sleep, happy, surprised, and wink), (right-light, left-right, center-light), and occlusion (with/without glasses). To test the robust of the proposed method, we conduct no preprocessing on the images.

We use a subset of the FERET database [29] including 400 images of 200 individuals. Each person has two images (fa and fb) which are obtained at different times and with different facial expressions. The images are cropped to the size of 128 by 128.

In the experiments on ORL and Yale databases, we use the first image of each individual for training and the rest images for testing. The training sets consist of 40 and 15 images in these two experiments, and their corresponding testing sets consist of 360 and 150 images. In the FERET database, we use the 200 fa images for training and the 200 fb images for testing.

### 1 Feature Extraction Methods

Besides the conventional PCA, LDA, and LPP, we compare our methods with other three methods [12], [19], [21] which are proposed to solve the one sample problem. The (PC)^2^A [10] is a PCA-based method and the methods in [19], [21] are LDA-based methods. The parameters of these three methods are set the same as those in [12], [19], [21], respectively. Additionally, we also compare our method with a LPP-based method which is referred to as projection-combined locality preserving projection (PCLPP) in this paper. This LPP-based method first enriches the face images using the method in [12] then implements the LPP method on the enriched images.

To extract discriminative features, we first enlarge the training set using Algorithm 1 and perform feature extraction on the enlarged training set. These methods are referred to as PCA on the enlarged training set (PCAoE), LDA on the enlarged training set (LDAoE), and LPP on the enlarged training set (LPPoE). The extracted features are classified using K-nearest neighbor (KNN) classifier.

Two important parameters in algorithm are: the number of neighbors 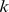 in step 1 and the parameter 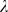 for interpolation in step 2. Table 1 presents the value of 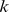 in these three databases. Table 1 shows that 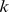 increases as the number of individuals increase. In step 2 of Algorithm 1, when synthesizing sample based on 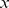 and its 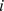
th nearest neighbor, the parameter 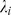 is required to be larger than 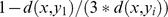 and no larger than 1. In our experiments, we set the parameter 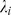 as follows
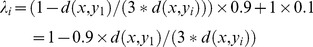
(31)where 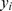 is the 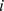
th nearest neighbor of 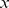. Based on equation (31), we know 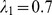 and 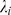 increases as the 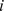 increases. Thus, the parameter is always larger than 0.7 in step 2.

**Table 1:** The parameters on the three databases.

database	ORL	Yale	FERET
Number of individual	40	15	200
*k*	9	7	21

**Figure 2:**
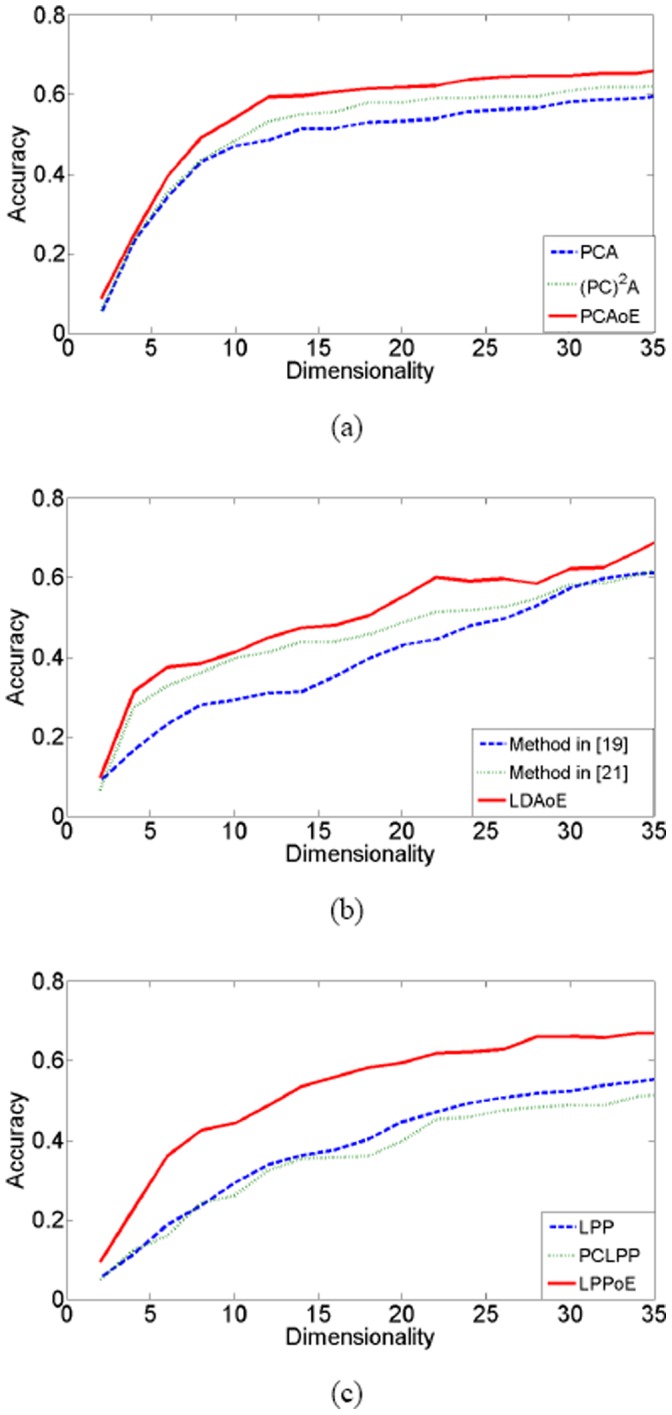
The experimental results on the ORL database.

**Figure 3:**
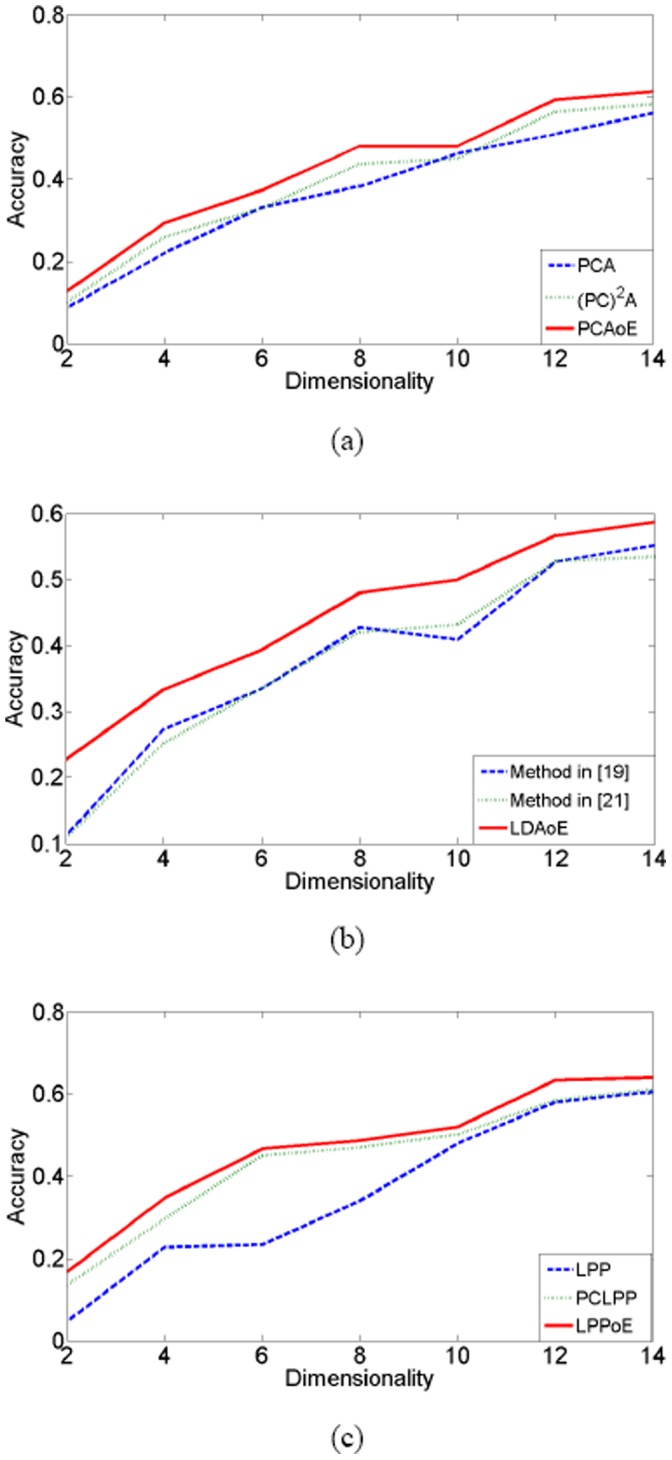
the experimental results on Yale database.

**Figure 4:**
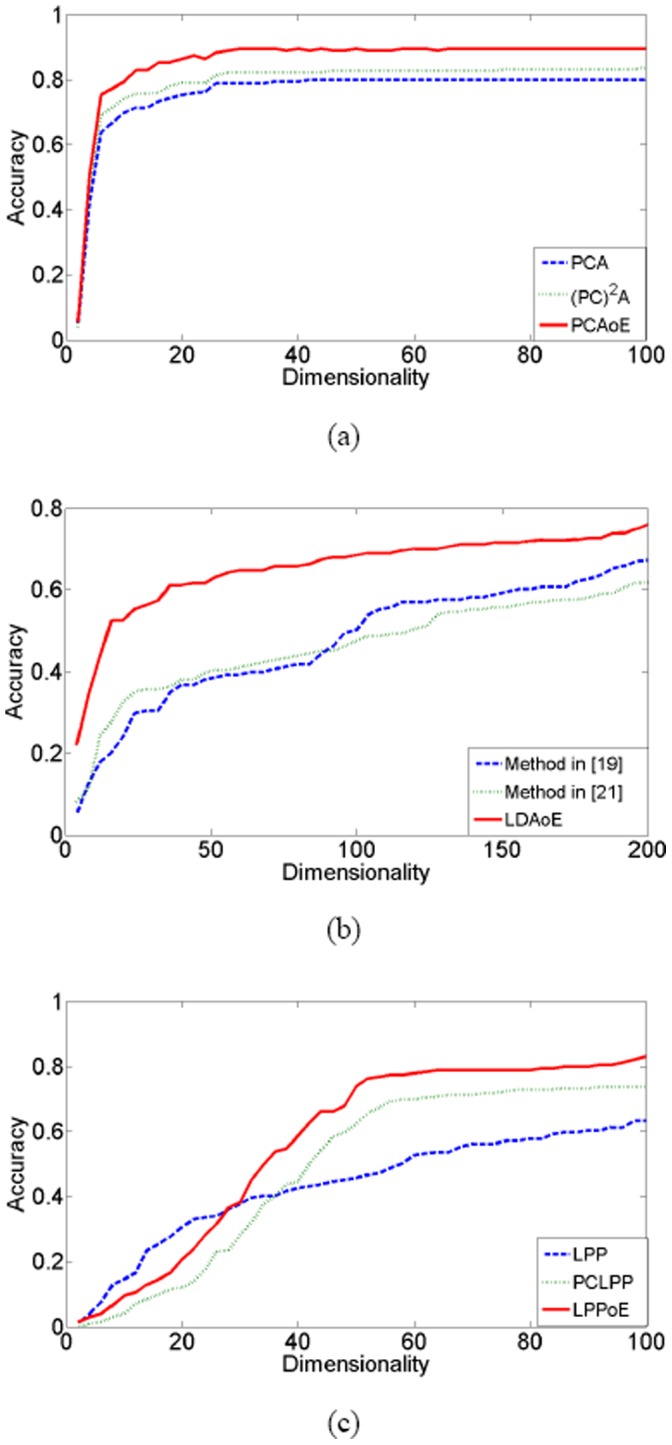
The experimental results on FERET database.

The figures 2, 3, 4 show the classification accuracy of different methods under different number of feature extractors on the three databases. As can be seen from these figures, the feature extraction methods achieve the highest classification accuracy if they are performed on the enlarged training set. Table 2 lists the highest classification accuracy of these methods. On the ORL database, the classification accuracy of PCAoE is 6.6% and 4.3% higher than those of PCA and (PC)^2^A; the classification accuracy of LDAoE is 9.5% and 8.3% higher than those of the methods in [19] and [21]; the classification accuracy of LPPoE is 11.2% and 15.5% higher than those of LPP and PCLPP. On the Yale database, the classification accuracy of PCAoE is 5.3% and 3.0% higher than those of PCA and (PC)^2^A; the classification accuracy of LDAoE is 3.5% and 5.3% higher than those of the methods in [19] and [21]; the classification accuracy of LPPoE is 3.3% and 2.9% higher than those of LPP and PCLPP. On the FERET database, the classification accuracy of PCAoE is 9.5% and 5.8% higher than those of PCA and (PC)^2^A; the classification accuracy of LDAoE is 8.6% and 4.2% higher than those of the methods in [19] and [21]; the classification accuracy of LPPoE is 19.8% and 9.2% higher than those of LPP and PCLPP.

**Table 2:** The highest classification accuracy (%) of different methods.

	PCA-based method			LDA-based method			LPP-based method		
	PCA	(PC)^2^A	PCAoE	Method in [19]	Method in [21]	LDAoE	LPP	PCLPP	LPPoE
ORL	59.9	62.2	66.5	61.3	62.8	70.8	55.8	51.5	67.0
Yale	56.0	58.3	61.3	55.2	53.4	58.7	60.7	61.1	64. 0
FERET	80.0	83.7	89.5	67.3	61.7	75.9	63.3	73.9	83.1

In our experiments, the original training sets of the ORL, Yale, and FERET databases consist of 40, 15, and 200 images, respectively. The training sets enlarged using algorithm 1 are much larger, and they consist of 400, 120, and 4400 images, respectively. Let real training image 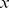 and testing image 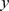 are images of the same individual. In our experiments, 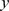 can be far from 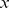 in the feature space, and a misclassification occurs. However, some certain synthesized variations of 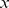 are neighbors of 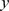. Then, 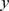 is correctly classified based on these neighbors. In this way, we can improve the classification accuracy significantly. This is especially true on the FERET database.

### 2 Sparse Representation

Recently, the sparse representation based classification (SRC) is widely studied recently and achieve high recognition accuracy with multiple training images from each person [9]. SRC can also work with a single training image. Here, we analyzed SRC to explore its ability in face recognition with a single training image and improve the accuracy with the enlarged training set. Though SRC can achieve very high accuracy when the training set consists of many images for each individual, it fails to do so in one sample problem. However, one image cannot capture the variations of the face images under different environments. For a test image, a number of training images from the same person can linearly express it with a small residue in terms of L2-norm. Thus, the linear expression of a test sample using all the training samples can be sparse. However, a single image cannot well express a test sample with a small residue. Thus, the sparse representation of a test sample using all the training samples normally has a large residue. Due to this, the sparsity of the coefficient is no longer discriminative enough. And the enlarged training set enriches the variations of the training set and enhances its representation. This significantly reduces the residue and enhances the discriminative of the coefficient in our experiment. Our method is feasible to increase the classification accuracy of SRC when the training set is very small. In this experiment, the training and testing set are the same as those above. We use SRC [9] to classify the testing samples first based on the original training set, then based on the training set enlarged using algorithm 1. Table 3 lists the classification accuracy of SRC based on the original and enlarged training set.

**Table 3:** The classification accuracy (%) of SRC three face databases.

	ORL	Yale	FERET
Original training set	61.3	46.0	83.9
Enlarged training set	65.5	54.0	86.4

Table 3 shows that the classification accuracies of SRC are normally lower than the highest classification accuracy of the PCA, LDA, and LPP-based methods. On the enlarged training set, SRC achieves higher classification accuracy. This is because the enlarged training set more representative than the original training set and can express the testing images more accurately. In our experiments, the coefficient of the linear expression is not as sparse as those in the multiple samples problems, as shown in [9].

### Conclusion

Most face recognition techniques require multiple images from each individual for training. The one sample problem either degrades the performance of these techniques or makes them fail to work. In this paper, we analyze the principal of three popular feature extraction methods (PCA, LDA, and LPP) and show why they cannot perform well on one sample problem. Moreover, we present analyses from a new viewpoint: why is one sample problem itself difficult? We ascribe the difficulty to four reasons: the SSS problem; the lack of representative samples; the underestimated intra-class variation; and the overestimated inter-class variation.

Based on our analysis, we propose a method to synthesize images and enlarge the training set for face recognition from one image per person. The synthesized images are weighted combinations of the pairs of real images. Two properties of the enlarged training set proclaim that the enlarged training set can replace the original training set. The enlarged training set overcomes the previously mentioned four difficulties of the one sample problem and improves the classification accuracy in our experiments.
